# Gene expression profiling of the synergy of 5-aza-2^′^-deoxycytidine and paclitaxel against renal cell carcinoma

**DOI:** 10.1186/1477-7819-10-183

**Published:** 2012-09-06

**Authors:** Tiandong Han, Donghao Shang, Xiuhong Xu, Ye Tian

**Affiliations:** 1 Department of Urology, Beijing Friendship Hospital, Capital Medical University, 95 Yong-An Road, Beijing, 100050, China

**Keywords:** Synergy, 5-aza-2^′^-deoxycytidine, Paclitaxel, Renal cell carcinoma, PI3K/Akt

## Abstract

**Background:**

Renal cell carcinoma (RCC) is one of the most common kidney cancers and is highly resistant to chemotherapy. We previously demonstrated that 5-aza-2^′^-deoxycytidine (DAC) could significantly increase the susceptibility of renal cell carcinoma (RCC) cells to paclitaxel (PTX) treatment *in vitro*, and showed the synergy of DAC and PTX against RCC. The purpose of this study is to investigated the gene transcriptional alteration and investigate possible molecular mechanism and pathways implicated in the synergy of DAC and PTX against RCC.

**Methods:**

cDNA microarray was performed and coupled with real-time PCR to identify critical genes in the synergistic mechanism of both agents against RCC cells. Various patterns of gene expression were observed by cluster analysis. IPA software was used to analyze possible biological pathways and to explore the inter-relationships between interesting network genes.

**Results:**

We found that lymphoid enhancer-binding factor 1 (LEF1), transforming growth factor β-induced (TGFBI), C-X-C motif ligand 5 (CXCL5) and myelocytomatosis viral related oncogene (c-myc) may play a pivotal role in the synergy of DAC and PTX. The PI3K/Akt pathway and other pathways associated with cyclins, DNA replication and cell cycle/mitotic regulation were also associated with the synergy of DAC and PTX against RCC.

**Conclusion:**

The activation of PI3K/Akt-LEF1/β-catenin pathway could be suppressed synergistically by two agents and that PI3K/Akt-LEF1/β-catenin pathway is participated in the synergy of two agents.

## Background

Renal cell carcinoma (RCC), a glandular carcinoma, accounts for approximately 85% to 95% of adult malignant kidney cancer cases [1]. Although surgical resection can be curative for localized disease, prognosis of advanced renal cell carcinoma is very poor with a five-year survival rate of 5% to 10%. At present, no standard treatment has been established for metastatic RCC, due to its high resistance to conventional chemotherapy [2,3]. The response rates of immunochemical therapies combined with chemotherapeutic agents with interferon-α or interleukin-2 ranged from 2% to 39% [4,5].

The landscape for RCC treatment has changed dramatically in recent years, vascular endothelial growth factor (VEGF) receptor tyrosine phosphorylation inhibitors (TKIs) and drugs that inhibit mammalian target of rapamycin (mTOR) signaling have become the mainstay for the management of metastatic RCC based on improved progression-free survival or/and overall survival outcomes [6]. As new targeted strategies to control renal cell carcinoma evolve, so do the strategies to measure response and predict outcome. Recently, more efforts had focused on exploring the critical role of DNA methylation in human carcinogenesis [7]. 5-Aza-2^′^-deoxycytidine (DAC), a nucleoside analogue, could incorporate into DNA and exert direct cytotoxic and antiproliferative effects on tumor cells [8]. These effects are mainly dependent on its interference with DNA reparative machinery and inhibition of de novo thymidine synthesis, as well as activation of proapoptotic intracellular signaling [9]. The ability of DAC might be attributed to its inhibition of DNA methylation and activation of cell cycle checkpoint signaling, similarly to previous reports for DNA repair responses [10,11]. These two activities may not be entirely independent of each other given that the expression of some genes involved in cell cycle regulation is epigenetically controlled.

Paclitaxel (PTX) is now considered a new type of broad-spectrum and highly efficient anticancer drug, and the efficacy of this agent on a variety of solid tumors has been noted. PTX is an anticancer agent due to its efficient induction of apoptosis. It interferes with microtubule assembly by binding and stabilizing b-tubulin in the G2/M phase of the cell cycle [12]. We have previously examined the antiproliferative effects of DAC alone, and of DAC with the various chemotherapeutic agents, on RCC cells. Our results showed that DAC combined with PTX synergistically inhibited the growth of the RCC cell lines [13]. We also investigated the basic mechanism of the synergy of DAC and PTX against RCC cells; DAC inhibited cell growth by the induction of G2/M cell cycle arrest, and the effect of PTX depended on apoptosis induction and G2/M cell cycle arrest. When treated with DAC and PTX together, a higher percentage of cells in subG1 and G2/M phase was observed compared to that in cells treated with DAC or PTX alone. Although caspase inhibitors could decrease PTX-induced apoptosis and the cytotoxicity of PTX in RCC cells, they did not abolish the enhancement of the susceptibility of RCC to PTX by DAC via G2/M cell cycle arrest. However, the molecular mechanism and pathways involved in the synergistic effect of these two agents against RCC remain unclear.

In this study, we investigated the gene transcriptional alteration by cDNA microarray and investigated possible molecular mechanism and pathways implicated in the synergy of DAC and PTX against RCC. Our results indicated that several key regulatory genes and active pathways could be identified and they may play critical roles in the synergy of DAC and PTX.

## Methods

### Cell culture and agents

Two RCC cell lines (human cells), ACHN and NC 65, obtained from ATCC were cultured in RPMI-1640 medium (Gibco, Bio-Cult, Glasgow, Scotland, UK) supplemented with 25 mM HEPES, 2 mM L-glutamine, 1% nonessential amino acids, 100 units/ml penicillin, 100 μg/ml streptomycin, and 10% heat-inactivated fetal bovine serum. Cell lines were maintained as monolayers in 10 cm plastic dishes and incubated in a humidified atmosphere containing 5% CO_2_ at 37°C. Cells were treated for three days. DAC and PTX were purchased from Sigma-Aldrich, St Louis, MO, USA.

### RNA purification and cDNA preparation

ACHN and NC 65 cells were treated with DAC (1 μM), PTX (1nM), DAC (1 μM) + PTX (1nM), or vehicle respectively. Total RNAs are harvested using TRIzol (Invitrogen, Carlsbad, CA, USA) and the RNeasy kit (Qiagen, Frankfurt, Germany) according to the manufacturer’s instructions. 1 μg total RNA was used as starting material for the cDNA preparation. After having performed RNA measurement on the NanoDrop ND-1000 (NanoDrop Technologies, Wilmington, DE, USA) and denaturing gel electrophoresis, the samples were amplified and labeled using the Agilent Quick Amp labeling kit (Agilent Technologies, Santa Clara, CA, USA), utilizing Cy5 and Cy3 fluorescent dye.

### cDNA microarray

Hybridization with Agilent’s whole genome oligo microarray was performed in Agilent’s SureHyb hybridization chambers. After hybridization and washing, the processed slides were scanned with the Agilent DNA microarray scanner (part number G2505B) using settings recommended by Agilent Technologies, and then quantitative analysis was conducted. The resulting text files extracted from Agilent Feature Extraction software (version 10.5.1.1) were imported into the Agilent GeneSpring GX software. Finally, genes that were upregulated or downregulated more than 2.0-fold in comparison with the control were selected and analyzed for further study.

### Real-time PCR

Eighteen upregulated and downregulated genes were chosen for verification by real-time PCR. PCR reaction was carried out by Platinum SYBR Green qPCR SuperMix UDG kit (Applied Biosystems, Foster City, CA, USA ). GAPDH gene was used as internal control. The PCR thermal cycling was done by a Bio-Rad real-time PCR machine (Bio-Rad Laboratories, Richmond, CA, USA), and conditions performed for all of the samples was as follows: samples were subjected to 40 amplification cycles comprising denaturation at 95°C for 10 sec, annealing at 60°C for 10 sec, and elongation at 72°C for 10 sec. Total RNA was isolated with RNeasy mini kit (Qiagen) and the first-strand cDNA synthesis kit (Amersham Biosciences, Little Chalfont, UK) was used for reverse transcription. Data collection was performed during both annealing and extension, with two measurements at each step and at all times during melt curve analysis.

### Western blot

We changed the treatment concentrations in this section. Protein was extracted by lysis buffer, the protein concentration was assessed by the Bradford dye-binding protein assay (Bio-Rad), and then SDS polyacrylamide gel electrophoresis was performed. Antibodies to PI3 Kinase p85 (19 H8)/phospho-PI3K p85 (Tyr458) Rabbit mAb; Akt, (pan) (11E7)/phospho-Akt (Ser473) Rabbit mAb were purchased from Cell Signaling Technology (Danvers, MA, USA). An anti-β-actin monoclonal antibody (Abcam, Cambridge, UK) was used as an internal control. The immune complexes were detected using a system of enhanced chemiluminescence (ECL) combined with Western blot (Amersham, Aylesbury, UK).

### Ingenuity pathway analysis

Ingenuity pathway analysis (IPA) version 3.0 was used to cluster genes for possible biological pathways and to explore the inter-relationships between interesting network genes with particular patterns. To start building networks, the program queries the Ingenuity Pathway Knowledge Base for interactions between Focus Genes and all other gene objects stored in the knowledge base, to generate a set of networks. IPA then computes a score for each network according to the fit of the network to the setoff focus genes. A detailed description of IPA can be found on http://www.ingenuity.com. All up/down-regulated genes (>2.0-fold) were picked up and analyzed by IPA to reveal the key functions and pathways that participated in the treatment of RCC by DAC and PTX.

### Statistical analysis

All determinations were repeated in triplicate, and the results were expressed as mean ± standard deviation (SD). Statistical significance was determined by Student’s t-test, and a *P* value of 0.05 or less was considered significant. Calculations of synergy were made by isobolographic analysis, as described by Berenbaum [14].

## Results

### cDNA microarray and cluster analysis

**Figure 1:**
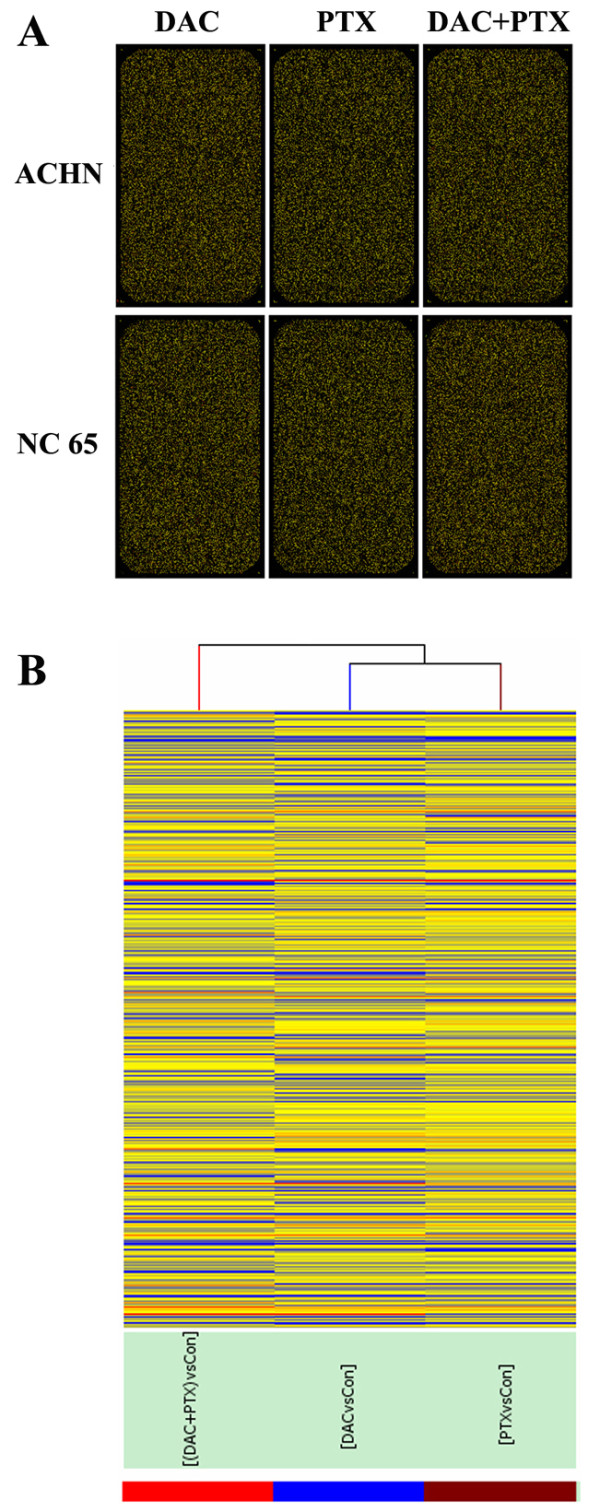
**Scanned images of cDNA microarray are displayed in (A) and (B) and the result of hierarchical clustering on conditions shows a distinguishable gene expression profiling among samples.** ‘Red’ indicates high relative expression, and ‘blue’ indicates low relative expression.

Scanning images of cDNA microarray are displayed in Figure 1A and 1B, Cy3 stands for the controls (green), whereas Cy5 stands for treatment with DAC, PTX or DAC + PTX (red). Hierarchical clustering was used to produce gene or condition trees. The resulting trees grouped genes (samples/conditions) together based on the similarity of their expression profiles, which allow the users to select groups with similar genes (samples/conditions). The dendrogram shows the relationships among the expression levels of conditions. Here, hierarchical clustering was performed based on ‘all targets value’. Our experiment consists of three different conditions. The result of hierarchical clustering on conditions shows a distinguishable gene expression profiling among samples. The confirmation of the repeatability of the synergy of DAC and PTX against RCC cells is shown in Additional file 1.

### Regulated genes by DAC and/or PTX in RCC

The top 10 up/down-regulated genes by the treatment with DAC, PTX or DAC + PTX normalized against the control were showed in Additional file 2: Table S1. The expression of each gene in the different treated samples was averaged and depicted as fold changes in comparison with the control. The threshold value used to screen up- or down-regulated genes was set as absolute value of log2 ratio ≥1.0 (fold change ≥2.0). Microarray data were normalized by dividing spot intensities by the global median. Normalized data were extracted, preprocessed and sorted with Microsoft Excel.

### Synergy-related genes of DAC and PTX

To identify the genes that were involved in the synergistic effect of DAC and PTX against RCC growth, we selected the genes that showed more than 2.0-fold changes in the samples treated with DAC, PTX and DAC + PTX than those in the control. The synergistic score presents the relativity level of the gene in the synergy of both agents, and a higher value indicates this gene may have participated in the effective interaction of these two agents. The synergistic score was calculated using the following formula: synergistic score = fold changes regulated by DAC + PTX/(fold changes regulated by DAC + fold changes regulated by PTX). The synergistic score of each gene was calculated by the average of ACHN and NC 65 and genes with the top 10 synergistic scores in this study are shown in Additional file 3: Table S2A and S2B.

### Synergy-related pathways of DAC and PTX

The IPA software’s output is ranked in terms of probability and the least likely to have occurred by chance will contain the largest number of disregulated genes and presumably be of the greatest interest and indicative of biologically relevant effects [15]. The canonical pathways involved in the synergy of DAC and PTX were shown as the *P* value, and low *P* value represents the pathway that is highly correlated with the synergy of DAC and PTX against RCC. We selected the four synergy-related pathways activated by DAC and/or PTX simultaneously in ACHN and NC 65 cells. These pathways include Class I PI3K signaling events mediated by Akt, amb2 integrin signaling, IL-2- and IL-23-mediated signaling events (Additional file 3: Table S2C). All of the four pathways could be activated by DAC and PTX alone. Moreover, a lower *P* value was achieved by combined treatment with DAC and PTX.

### Confirmation of synergy-related genes

**Figure 2:**
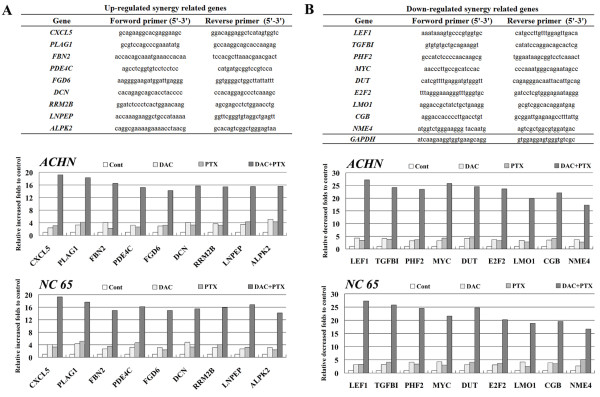
Confirmation of synergy-related genes of DAC and PTX by real-time PCR and primer sequences (A, B).

To confirm the repeatability of microarray data, nine upregulated and nine downregulated synergy-related genes were verified by real-time PCR. The primer sequences used in this study are listed in Figure 2, and results indicated that the expression of all 18 genes displayed similar synergistic score patterns to those identified in the original microarray data.

### Suppression of PI3K/Akt pathway by DAC and/or PTX

**Figure 3:**
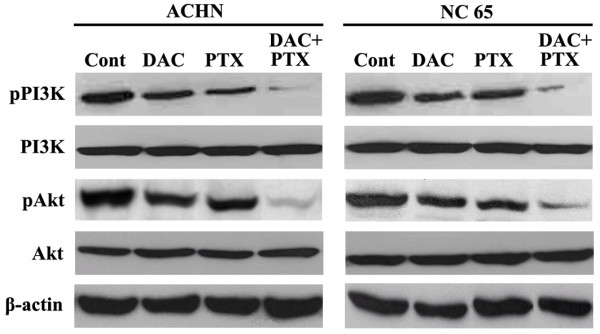
DAC and PTX suppressed the activation of the PI3K/Akt pathway significantly compared to DAC or PTX alone by Western blot.

To clarify how PI3K/Akt pathway is involved in the synergy of DAC and PTX against RCC cells, the phosphorylation of PI3K/Akt was evaluated after stimulation by DAC and/or PTX. In two RCC cell lines, although DAC (2 μM) and/or PTX (2 nM) did not affect the total expression of PI3K or Akt, both DAC and PTX alone decreased the phosphorylation of PI3K and Akt. Moreover, DAC significantly enhanced the suppression of phospho-PI3K and phospho-Akt induced by PTX in two RCC cell lines (Figure 3). These results suggest that PI3K/Akt pathway may play a key role in the synergy of DAC and PTX against RCC cells.

## Discussion

A large number of basic experiments and clinical trials of combination chemotherapy regimens have been performed with the hope of removing the limitations of current therapies for RCC. However, few of them have attained a remarkable response and prognostic benefit to patients [16]. Therefore, effective regimens of combination chemotherapy for RCC are highly sought.

Promising new antitumor agents usually appear as our understanding of oncogenesis advances. Combination chemotherapy of DAC and chemotherapeutic agents have been investigated since 2004, the results suggesting that DAC could increase the cytotoxicity of chemotherapeutic agents against lung cancer cells and melanoma cells *in vitro*[17,18]. In the previous study, we also reported the synergistic growth suppression of DAC with PTX in RCC [13]. DAC is a demethylation agent, which was shown to suppress the proliferation of malignant tumors by reactivating the expression of specific methylated genes or causing genome-wide demethylation [19,20]. However, another study suggested that DAC-induced antineoplastic activity was dependent on DNA damage [21]. Whether DAC acts on tumors primarily through its effect on DNA methylation or through synergistic cytotoxicity with PTX remains unknown.

In this study, we investigated the gene transcriptional alteration by the cDNA microarray and revealed possible molecular mechanism and pathways implicated in the synergy of DAC and PTX against RCC cells. Several key regulatory genes were identified and may play critical roles in the synergy of these two agents. These include lymphoid enhancer-binding factor 1 (LEF1), transforming growth factor β-induced (TGFBI), C-X-C motif ligand 5 (CXCL5) and myelocytomatosis viral related oncogene (c-myc). LEF1 was initially identified as a pre-B and T lymphoid-specific gene encoding a DNA-binding protein of high mobility group proteins [22,23]. In addition, LEF1 is a member of the lymphoid-enhancing factor/T-cell factor (LEF/TCF) family of transcription factors, which acts through the Wnt/β-catenin signaling pathway to regulate gene expression and coordinate many cellular processes in normal development and carcinogenesis [24-26]. A study showed that the proliferation and invasion of the melanoma cell is regulated by LEF1/TCF activity [27]. Upon Wnt stimulation, LEF1 could combine with β-catenin and activate Wnt-responsive target genes [28]. Our previous study has confirmed that LEF1 can enhance the proliferation of RCC cells and that suppressing the expression of LEF1/β-catenin complex plays an important role in the synergistic mechanism of DAC and PTX against RCC cells [29]. TGFBI is a target of TGF-β and secreted into the extracellular space, where it binds to fibronectin and collagen as well as integrins to stimulate adhesion, migration, spreading, and proliferation in renal proximal tubular epithelial cells [30,31]. Studies showed that TGFBI was induced by TGF-β in the lung adenocarcinoma cell line and overexpression of TGFBI was associated with some malignancies, such as RCC and hemangioblastoma [32,33]. Our previous study has demonstrated that TGFBI-promoted metastasis of RCC cells depends on inactivation of the von Hippel-Lindau (VHL) tumor suppressor and that TGFBI could be a therapeutic target against RCC in the future [34]. CXCL5, a member of the CXC chemokine family, has been shown to be involved in angiogenesis, tumor growth, and metastasis. CXCL5 is upregulated significantly in sporadic endometrioid endometrial adenocarcinomas compared to normal endometrium [35]. CXCL5 overexpression was also associated with late stage gastric cancer and high N stage, suggesting CXCL5 is involved in the progression of gastric cancer, especially in lymph node metastasis [36]. However, whether CXCL5 could stimulate phenotypic responses in renal epithelial cells with malignant progression remains unknown. c-myc is a multifunctional, nuclear phosphoprotein that plays multiple roles in eukaryotic cells including cell progression, differentiation, apoptosis and neoplasia [37]. It interferes with the regulators of G1/S transition, as well as other regulators of cell growth and metabolism, inducing several translation factors and adhesion molecules [38]. Alterations of the c-myc genomic region are usually observed in prostate cancer [39-41] and bladder cancer [42], however, genomic alterations of c-myc are mostly subordinate for conventional RCC with the exception of papillary renal cancer [43-45].

At present, the signaling pathways underlying the synergy of DAC and PTX against RCC have not been investigated. In this study, we found that some cell cycle-related pathways were involved in the synergy of both agents by IPA, such as cyclins, DNA replication and cell cycle/mitotic regulation; these results are consistent to our previous study. Moreover, we also confirmed that the synergy of DAC and PTX may be mediated by inhibiting the PI3K/Akt pathway. PI3K could enhance production of PIP3 and trigger a signaling cascade, which results in the activation of Akt [46]. Because LEF1/β-catenin is a target of the PI3K/Akt pathway, we speculated that synergistic suppression of LEF1/β-catenin expression by DAC and PTX is dependent on the inactivation of the PI3K/Akt pathway.

## Conclusion

In conclusion, we analyzed the overall gene expression profiling by cDNA microarray and revealed the synergistic mechanism of DAC and PTX against RCC. Our results indicated that activation of the PI3K/Akt-LEF1/β-catenin pathway could be suppressed synergistically by these two agents and that the PI3K/Akt-LEF1/β-catenin pathway participated in the synergy of the two agents. More altered genes and pathways involved in the synergy should be investigated in the future.

## Competing interests

The authors declare that they have no competing interests.

## Authors’ contributions

TH, DS conceived and designed the experiments. TH, XX performed the experiments. DS analyzed the data. DS, XX contributed reagents/materials/ analysis tools. TH, YT drafted the manuscript. All authors read and approved the final manuscript.

## Supplementary Material

Additional file 1 **DAC (A) and PTX (B, C) each caused dosage-dependent cell growth suppression of RCC cells.** DAC (0.5 and 1 μM) increased the susceptibility of RCC cells to PTX (B for ACHN and C for NC 65 are shown). The combination of DAC and PTX caused synergistic growth suppression in all two RCC cell lines by isobolographic analysis (D). Click here for file

Additional file 2 Table S1 The top 10 up/down-regulated genes in the three different conditions normalized by untreated control. Click here for file

Additional file 3 Table S2 The top 10 up/down-regulated synergy-related genes by DAC and PTX are shown in (A) and (B), and synergy-related pathways by DAC and/or PTX are shown in (C). Click here for file
