# Supplementary material for: Gene expression profiling of the synergy of 5-aza-2′-deoxycytidine and paclitaxel against renal cell carcinoma
Source: World J Surg Oncol. 2012 Sep 6;10:183. doi: 10.1186/1477-7819-10-183 (PMC3481426; doi:10.1186/1477-7819-10-183)
Supplement: Additional file 1 — DAC (A) and PTX (B, C) each caused dosage-dependent cell growth suppression of RCC cells. DAC (0.5 and 1 μM) increased the susceptibility of RCC cells to PTX (B for ACHN and C for NC 65 are shown). The combination of DAC and PTX caused synergistic growth suppression in all two RCC cell lines by isobolographic analysis (D). [file 1477-7819-10-183-S1.doc]

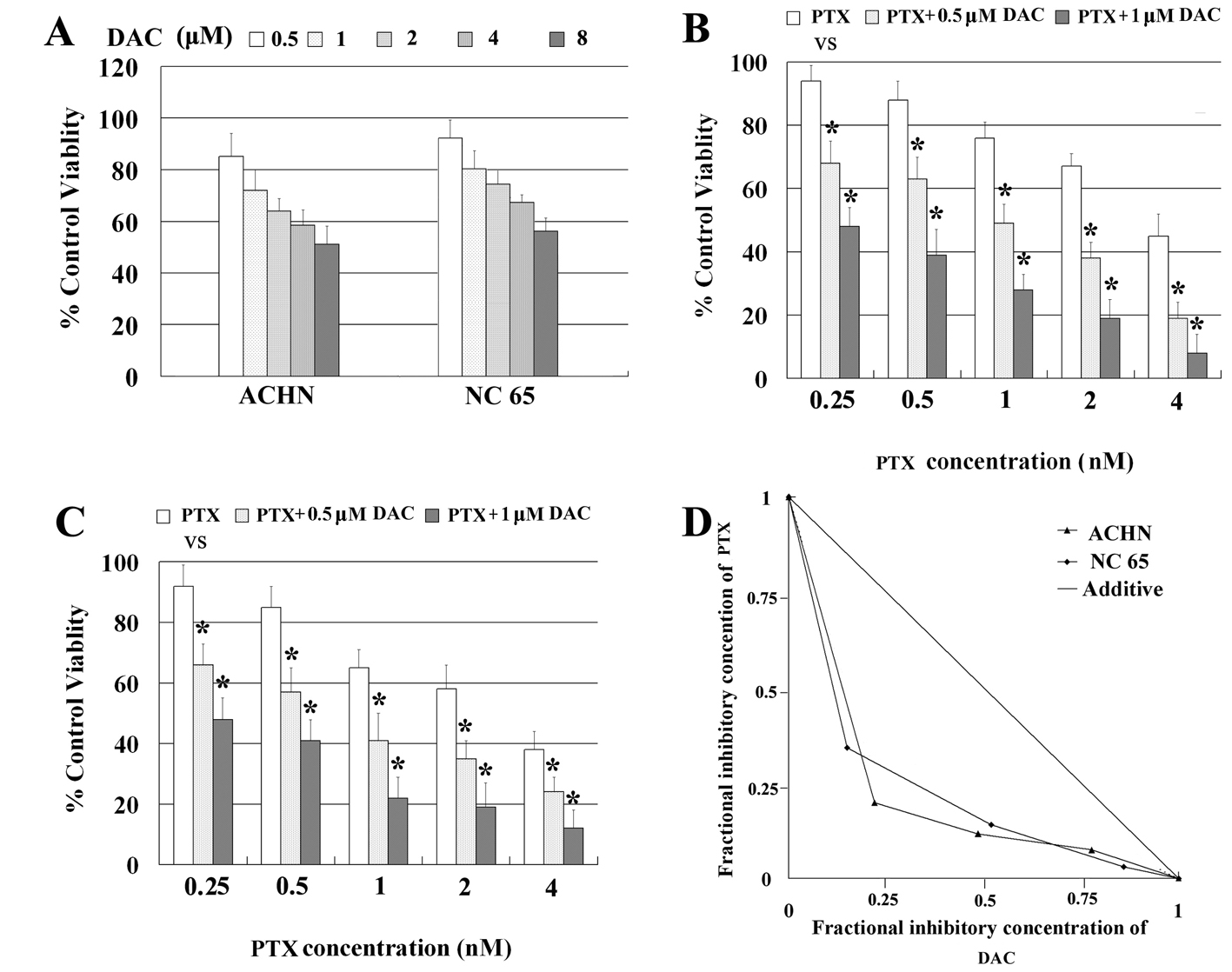


### DAC (A) and PTX (B, C) each caused dosage-dependent cell growth suppression of RCC cells. DAC (0.5 and 1 μM) increased the susceptibility of RCC cells to PTX (B for ACHN and C for NC 65 are shown). The combination of DAC and PTX caused synergistic growth suppression in all two RCC cell lines by isobolographic analysis (D).
