# Supplementary figures and images for: Gene expression profiling of the synergy of 5-aza-2′-deoxycytidine and paclitaxel against renal cell carcinoma
Source: World J Surg Oncol. 2012 Sep 6;10:183. doi: 10.1186/1477-7819-10-183 (PMC3481426; doi:10.1186/1477-7819-10-183)

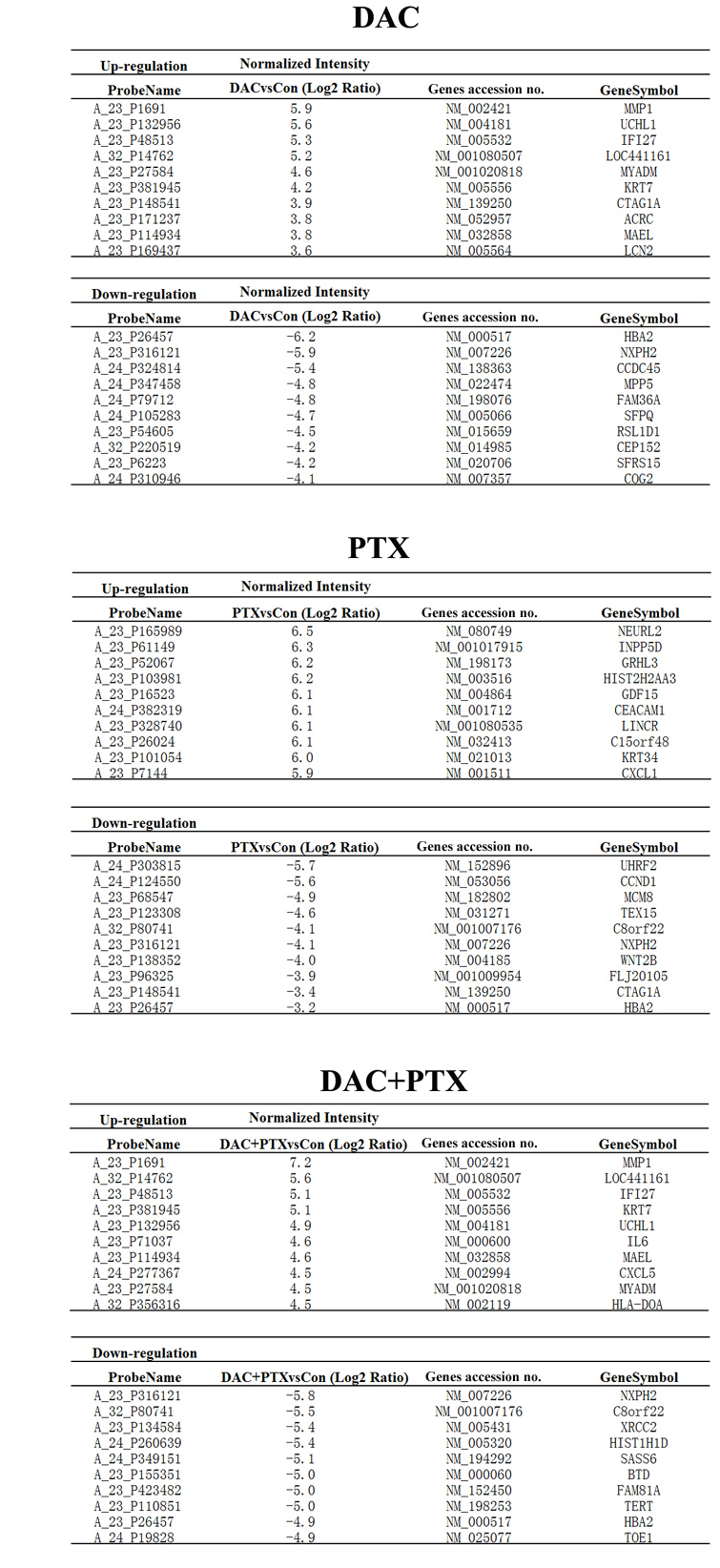

Supplement: Additional file 2 — Table S1 The top 10 up/down-regulated genes in the three different conditions normalized by untreated control. [file 1477-7819-10-183-S2.jpeg]

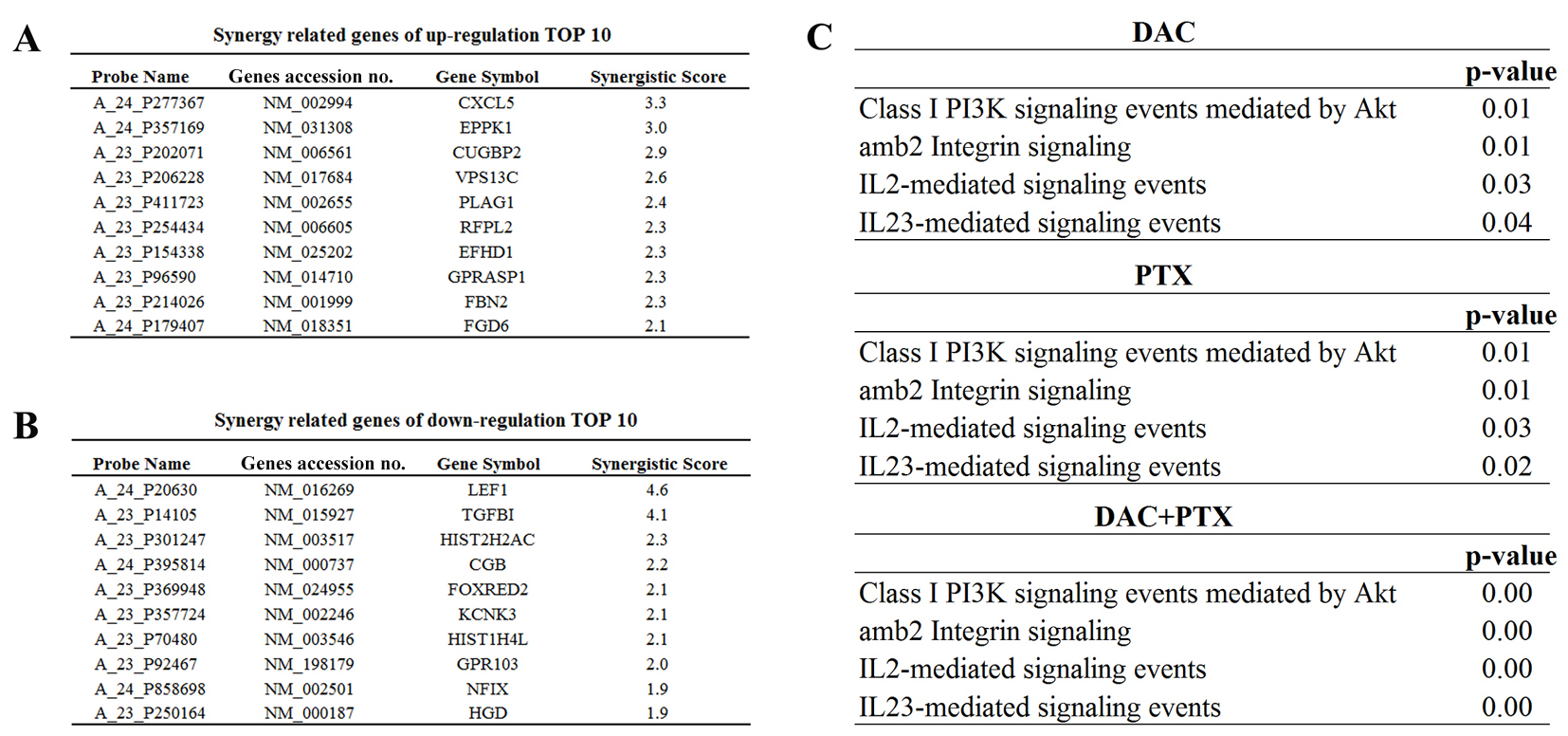

Supplement: Additional file 3 — Table S2 The top 10 up/down-regulated synergy-related genes by DAC and PTX are shown in (A) and (B), and synergy-related pathways by DAC and/or PTX are shown in (C). [file 1477-7819-10-183-S3.jpeg]
